# Prevalence and concentration of pesticides in European waters: A protocol for systematic review and meta-analysis

**DOI:** 10.1371/journal.pone.0282386

**Published:** 2024-03-26

**Authors:** Manuel Serrano Valera, Nuria Vela, Grasiela Piuvezam, Francisco Mateo-Ramírez, Isac Davidson Santiago Fernandes Pimenta, Isabel Martínez-Alcalá

**Affiliations:** 1 Catholic University of Murcia, Murcia, Spain; 2 Systematic Review and Meta-Analysis Laboratory (LabSys-CNPQ), Federal University of Rio Grande do Norte, Natal, Brazil; 3 Department of Public Health, Federal University of Rio Grande do Norte, Natal, Brazil; 4 Post-Graduation Program in Public Health, Federal University of Rio Grande do Norte, Natal, Brazil; Universidad San Francisco de Quito, ECUADOR

## Abstract

There is currently a growing interest in the so-called emerging pollutants, such as pesticides, pharmaceuticals, personal hygiene care products, drugs, etc., whose presence in natural ecosystems is not necessarily recent, but the development in latest years of new and more sensitive methods of analysis has allowed their detection. They can be present in the natural environment, food, and many products of everyday origin, which suggests that human exposure to them is massive and universal. Therefore, the study of this type of substances is becoming one of the priority lines of research of the main agencies dedicated to the protection of public and environmental health, such as the World Health Organization (WHO), United States Environmental Protection Agency (USEPA) or European Union (EU). In this sense, it is of vital importance to know the nature and quantity of this type of contaminants, to establish preventive mechanisms that minimize its presence in aquatic systems, with special requirements for human consumption. This study aimed to describe a protocol for a systematic review and meta-analysis to assess the status of pesticides in European waters. We will search for original studies in the PubMed/Medline, Scopus, Web of Science, EMBASE, ScienceDirect databases. Prevalence studies of emerging contaminants (pesticides) in water resources (watersheds, aquifers, rivers, marine and springs), wastewaters (influent and effluent), and drinking water should be included. Two reviewers will independently screen and assess the included studies, with any disagreements being resolved by a third reviewer. We will summarize the findings using a narrative approach and, if possible, conduct a quantitative synthesis (meta-analysis). We will conduct the protocol following the Preferred Reporting Items for Systematic Review and Meta-Analyses Protocols (PRISMA-P) guidelines. The review will summarize the current evidence on the presence of pesticides in European waters such as glyphosate, chlorpyrifos, pyrethroid pesticides, neonicotinoid pesticides, and/or fungicides, in samples of different water resources like wastewaters and drinking water. We expect that this systematic review will establish preventive mechanisms that minimize the presence of pesticides in water in the environment.

## Introduction

Water scarcity is a global issue that affects all continents and constitutes one of the major challenges of the 21st century. Over the past few years, water use and consumption have grown at twice the rate of population growth. Although there is enough drinking water on the planet, its distribution is irregular, contaminated in many cases, and is often unsustainably managed [1].

The supply problem is exacerbated by the significant pollution of surface water and groundwater due to discharges of waste from human activity. The United Nations estimates that 2 million tons of waste are disposed of through waterways every day [2]. Such pollution reduces the availability of safe drinking water and requires an extensive treatment process.

Although substantial pollution prevention measures implemented in the last century have drastically reduced the presence of some pollutants, such as the so-called "persistent organic compounds", the list of known potentially dangerous substances that can affect the environment, and thus humans, is extensive [3, 4]. Currente research attention are focused on the class of "emerging contaminants" (ECs). ECs are natural or synthetic chemical substances of various origins, and their presence in natural ecosystems is not necessarily new. However, the development of more sensitive analytical methods in recent years has made it possible to detect their presence, increasing concern about their potential consequences for human health [5].

ECs are found in many types of everyday chemicals, such as pesticides used to protect crops from insects, weeds, fungi and other pests [6]. This subgroup of emerging contaminants is of particular concern to agencies such as the World Health Organization (WHO) and the European Food Safety Authority (EFSA) [7–9]. Most of these substances are very persistent in the environment and can be highly toxic to humans. There is scientific evidence that chronic exposure to pesticides can lead to various health issues such as cancer, neurologic disorders, diabetes, respiratory disorders, and reproductive syndromes [10]. Moderate pesticide poisoning can cause intrinsic asthma, bronchitis, and gastroenteritis. Acute effects may include reduced vision, headaches, salivation, diarrhea, nausea, vomiting, wheezing, coma and even death [11]. These effects depend on the chemical nature of the compound, the amount, and the mode of exposure (ingestion, inhalation or direct skin contact) [12].

On the other hand, pesticides are very important for food production, as they maintain and/or increase crop yields, which is especially important in countries with food shortages. The need to produce enough food to feed the world’s population, combined with political and economic interests, has left an impact on agricultural practices worldwide. In many countries, particularly those with more flexible regulations, these pressures have led to expanded use of fertilizers and pesticides to achieve and maintain higher yields [13–15].

The European Union (EU) has established a framework for the prevention and control of water pollution, that includes measures to assess the chemical status of water and strategies to reduce the presence of pollutants. As stated in Directive 2013/39/EU of 12 August 2013 amending Directives 2000/60/EC and 2008/105/EC in relation to priority substances in the field of water policy [16], priority must be given to identifying the causes of pollution and addressing pollutant emissions at their source, in the most economically and environmentally effective way. Some strategies were agreed in the Millennium Development Goals through the Sustainable Development Goals (SDGs), especially Goal 6 "Ensure availability and sustainable management of water and sanitation for all" [17].

The EU recently revised the regulation on surface water and groundwater pollution, urban wastewater, and the sustainable use of pesticides [18–20]. This evidence that the study of these substances is research priority for the protection of public health and the environment [4].

Despite all these regulatory frameworks established by competent authorities, the presence of pesticide residues in aquatic ecosystems remains high. This underscores the need to understand the prevalence and extent of this type of contaminant, in order to formulate preventive strategies to minimize their presence in the environment, particularly in aquatic systems, specially water designated for human consumption [11, 21–23]. Thus, this paper describes a protocol for a systematic review and meta-analysis to assess the prevalence and concentration of pesticides in European waters.

## Methods and analysis

### Study registration

This protocol was registered in the International Prospective Register of Systematic Reviews (PROSPERO) database (CRD42022348332), and it is in line with international ethical parameters. Also, it was developed in accordance with the Preferred Reporting Items for Systematic Review and Meta-analysis Protocols (PRISMA-P) statement guidelines (S1 Checklist) [24].

The final report will be developed following PRISMA [25] and the Cochrane Handbook for Systematic Reviews of Interventions [26] and any changes to the protocol will be described in the Method section.

### Review question

The present protocol was conducted to answer the following questions that will correspond to a systematic review and meta-analysis: What is the prevalence and level of pesticide contamination in European waters?

### Eligibility criteria

#### Population

Studies that analyzed water resources (watersheds, aquifers, river, marine and springs), wastewaters (influents and effluents) and drinking waters from European countries.

#### Exposure

We will analyse the exposure to pesticides such as–but not limited to–glyphosate, chlorpyrifod, pyrethroid pesticides, neonicotinoid pesticides and/or fungicides.

#### Comparison

We will include studies that compare water resources (watersheds, aquifers, river, marine and springs), wastewaters (influents and effluents) and drinking waters from Europe to other countries or regions.

#### Outcome

The main outcome is the prevalence and concentration of pesticide residues in waters resources, wastewater, and drink waters.

#### Study types

We will include observational studies with a cross sectional, time series or cohort design.

#### Inclusion criteria

The articles selected for this review will be publications from 2015 onwards, as the EU Water Framework Directive (2000/60/EC) [16] requires all Member States to protect and improve water quality to achieve good ecological status by 2015. There will be no limitations in language in the searches performed.

#### Exclusion criteria

We will not consider: studies focused on leachates and stormwaters, experimental studies at laboratory scale, pilot plant conditions in water treatment plants, drinking water treatment, review studies, toxicity studies of disinfection by-products (DBPs), resistance mechanism studies, toxicological risk assessment studies in animals, human exposure risk studies, analytical methodology studies, emerging contaminants degradation pathway studies, and non-project studies.

Preprint studies, review articles, theses, dissertations, letters, conference abstracts, and gray literature will also be excluded.

### Information sources and search strategies

A comprehensive search will be elaborated from combinations of MESH and EMTREE terms combinations using Boolean operators (AND and OR) on databases, without time or language restrictions. The searches will be conducted in the following electronic bibliographic databases: PubMed, Scopus, EMBASE, Web of Science and ScienceDirect. The search equation for the systematic review will be defined considering the following terms for the PUBMED database: (“water resources” OR wastewater OR “drinking water” OR water OR sanitation AND pesticides AND prevalence OR monitoring AND Europe).

Adjustments may be necessary for the search strategy, considering the characteristics of the electronic databases, and terms may be included or changed. Additionally, a manual search will be performed in the references cited in the included articles to locate any additional relevant articles not retrieved within the primary search.

### Study selection

Initially, all the references will be screened for identification of duplicated records, followed by their proper removal. After this procedure, the articles will be read by two reviewers independently. First, the titles and abstracts of the works will screened. Subsequently, the selected articles will be read in full. Disagreements will be resolved by a third reviewer. All articles will be imported into the Rayyan app (version 0.1.0) [27]. The reasons for exclusion from studies will be recorded.

### Data extraction

Two reviewers will extract the data independently, using a standard electronic spreadsheet previously tested, and will include: reference data (aim, year of publication, main author, region of Europe, country, location); study design and population characteristics (type of water, sampling time, number of sampling sites, year of sampling); type of pesticide (number of analyzed compounds, chemical name of analyzed compounds, CASRN of analyzed compounds, family of compounds, methods of analysis detected) and it’s concentration (maximum, minimum and median values).

### Dealing with missing data

In the case of interesting data missing or being unclear, the research team will try to contact the corresponding author by email, phone or correspondence. If this communication is unsuccessful, we will exclude the data from the analysis, covering this in the Discussion section.

### Risk of bias and quality assessment

Two reviewers will independently assess the selected studies, using the Cochrane Risk Of Bias In Non-randomized Studies—of Exposure (ROBINS-E), a tool that provides a structured approach to assessing the risk of bias in observational epidemiological studies. ROBINS-E is designed primarily for use in the context of a systematic review, classifying the risk of bias as low, high or unclear [28].

In addition, the Grading of Recommendations Assessment, Development and Evaluation (GRADE) tool will be used to assess the quality of evidence [29]. To calculate the inter-rater reliability, we will use the kappa coefficient.

### Data synthesis

We will carry out a narrative synthesis and produce summarizing tables considering the data extraction plan. Based on the results of the analysis of the proportion of contaminated samples, clusters that define the frequency of the contamination will be created. In particular, the contamination for each pesticide will be defined as: Rare: the substance is present in < 1% of the samples; Relatively rare: the substance is present in 1–5% of the samples; Relatively common: the substance is present in 5–10% of the samples; Common: the substance is present in > 10% of the samples.

Dichotomous outcomes will be summarized as prevalence ratios or odds ratio with 95% confidence interval (CI), whereas continuous outcomes will be summarized as mean differences with 95% CI. The p value of each result will also be considered [30]. If the included studies are methodologically homogeneous, we will conduct a meta-analysis.

To verify the heterogeneity between studies, we will use the χ^2^ test with a significance of p<0.05. In addition, the I^2^ statistic will be calculated to assess the consistency between studies, considering a value of 0% as no observed heterogeneity, up to 50% as a moderate level and 75% or higher as a substantial level of heterogeneity. If possible, we will perform a funnel plot analysis to indicate possible reporting biases and a linear regression approach to measure the asymmetry of the funnel plot. Review Manager software (RevMan V5.3.3) will be used for the data analysis.

### Dissemination and ethics

We will publish the results of the systematic review in a peer-reviewed journal. In addition, the results will be disseminated in academic and health service spaces, such as at conferences or seminars. Because the systematic review will only use secondary data, there is no need to seek approval from a research ethics committee for this research.

## Discussion

Studies carried out in recent years reveal the presence not only of the well-known ECs but also of numerous variants of these families, in this case the study of pesticides. Pesticides can be found in the water bodies, derived above all, by the runoff from the agricultural field where they have been applied and industrial wastewaters. Especially pesticides more soluble are transported by water molecules during the precipitation event by percolating downward into the soil layers and eventually reaching surface waters and groundwater [31]. Consequently, it produces a degradation of water quality and then reduces the supply of potable water. The presence of pesticides in different environmental compartments, such as groundwater and surface water are a widespread problem. For example, pesticides with high mobility and persistence like organophosphates are detected most frequently in streams and groundwater around the world [32].

Although the use of pesticides is necessary to achieve higher agricultural yields, the occurrence of pesticides in water poses a deleterious effect on human and ecosystem health, where the effect magnitude depends on the pesticide properties such as solubility, half-life, adsorption capacity or biodegradability [33]. The effects produced in human beings can be acute or chronic. Some concrete examples of the effects of the pesticides above human health are reported in literature [10]. Frequent exposure to chlorinated pesticides had a high chance of inducing prostate cancer and allergic or non-allergic asthma in farmers [34].

To protect public and ecosystem health, guideline levels for pesticides in drinking waters have been restricted by governments. For example, there are several guideline values for the presence of pesticides in drinking water issued by WHO, United States, Australia, EU and Japan [35]. In fact, new legislations have been implemented in Europe for the use of regenerated water containing pesticides.

Concerns have increased, as has the need to detect them and use new techniques to reduce and eliminate them. For this reason, this study was undertaken because, to the authors knowledge, there are many site-specific monitoring studies, but no studies that conduct a systematic review and meta-analysis of pesticides in Europe.

The study focuses on the systematic review of the status of European Mediterranean waters through a meta-analysis of data collected in studies conducted in recent years using the most relevant databases in this research area. To establish comparative studies between the different regions of the Mediterranean and the repercussion of these pollutants by families. To offer not only a global vision of the current situation of pharmaceuticals, drugs and personal hygiene products, but also new possibilities to increase the detection and elimination systems of these ECs.

Therefore, it is necessary to resort to what are known as monitoring studies that, through the design and implementation of methods, processes and procedures, guarantee, to a large extent, the follow-up and evaluation of these pesticides, as well as the implementation or execution of observation systems for the ecosystems [36]. In this respect, chemical pollution of natural waters represents a threat to the environment, with effects such as acute or chronic toxicity in aquatic organisms, accumulation of pollutants in the ecosystem and loss of habitats and biodiversity [37, 38]. As stated in Directive 2013/39/EU, the causes of pollution must be identified, and pollutant emissions must be dealt with at source in the most economically and environmentally effective way [16]. There are no studies in the literature dealing specifically with this issue and the results of this study will help in the elaboration of public policies for the whole of Europe. The results obtained through this study will contribute to the development of public policies for Europe and probably for the whole world.

## Supporting information

S1 Checklist PRISMA-P checklist. (DOC)
